# Small Cell Lung Cancer Presenting as Severe Thrombocytopenia and Refractory Hypokalemia

**DOI:** 10.1155/2014/874831

**Published:** 2014-05-15

**Authors:** Rohan Mandaliya, Lesley Hughes, Herbert Auerbach, Felice LePar

**Affiliations:** ^1^Department of Internal Medicine, Abington Memorial Hospital, Abington, PA 19001, USA; ^2^Temple University School of Medicine, Philadelphia, PA 19140, USA; ^3^Department of Pathology, Abington Memorial Hospital, Abington, PA 19001, USA; ^4^Department of Hematology and Oncology, Abington Memorial Hospital, Abington, PA 19001, USA

## Abstract

A 70-year-old female with a history of mild cirrhosis was referred by her primary care provider for a platelet count of 36,000/**μ**L which had dropped from 47,000/**μ**L in a week along with mild pain in extremities. Serum potassium was low (2.9 mEq/L) in spite of the patient being recently started on potassium supplement on outpatient for hypokalemia. Initially thrombocytopenia was attributed to cirrhosis. However, platelet counts continued to drop to a nadir of 9000/**μ**L in spite of several platelet transfusions. Hypokalemia was refractory to potassium supplements. Subsequent bone marrow biopsy revealed extensive marrow necrosis with a focus of small cell tumor cells of pulmonary origin. CT scan of the chest showed a spiculated left lung mass. The ACTH level was high, with normal rennin and aldosterone levels. The patient likely had ectopic ACTH syndrome from small cell lung cancer. She died within few days of diagnosis. Severe thrombocytopenia and refractory hypokalemia can rarely be initial presentations of small cell lung cancer. Thrombocytopenia should prompt an evaluation for bone marrow metastases and a search for undiagnosed systemic malignancy. In severe cases of metastases, bone marrow necrosis can be present. Refractory hypokalemia can be the sole presentation of ectopic ACTH production.

## 1. Introduction

Small cell tumor of the lung is a neuroendocrine tumor and can be associated with paraneoplastic syndromes. Ectopic ACTH secretion (EAS) is a rare cause of Cushing's syndrome accounting for about 15% of cases [1]. Almost all tumors have been associated with EAS; however lung cancer (including small cell carcinoma and bronchial carcinoids) accounts for half of the cases and more than a quarter of the cases remain occult without determining the source of the ectopic secretion [1]. Refractory hypokalemia without any other metabolic abnormalities or cushingoid features can rarely develop due to ectopic ACTH production and rarely may be an initial presentation of the cancer. Thrombocytopenia may be the first presentation of a solid tumor such as lung cancer. Thrombocytopenia from malignancy can result either from bone marrow necrosis due to bone marrow metastasis or a paraneoplastic process like immune thrombocytopenic purpura, thrombotic thrombocytopenic purpura, myelodysplastic syndrome, or amegakaryocytic thrombocytopenia [2, 3]. Bone marrow necrosis is a rare clinicopathologic entity. The purpose of this case study is to (1) recognize the presentation of ectopic ACTH syndrome, (2) introduce the clinicopathologic entity of bone marrow necrosis and its implications, and (3) highlight various causes of thrombocytopenia associated with occult solid malignancies.

## 2. Case

A 70-year-old female visited her primary care for worsening fatigue with some proximal muscle weakness for two weeks. The patient had low back pain for the last two months due to T12 vertebral compression; however she had new onset of pain in her extremities for one week. The patient had a history of stable cirrhosis. There was no history of mucosal bleeding, epistaxis or easy bruising, or any recent viral illness. Laboratory testing as an outpatient revealed low platelet count of 47,000/*μ*L with hypokalemia of 2.9 mEq/L. She was started on oral potassium and referred to a hematologist for thrombocytopenia. Within one week the platelet counts dropped to 36,000/*μ*L. The patient was then admitted to the hospital. The vitals were heart rate of 80 bpm, blood pressure of 130/70 mm of hg, and respiratory rate of 16/min. There was mild proximal myopathy. There were no petechiae or rash. Otherwise the examination was benign.

**Figure 1:**
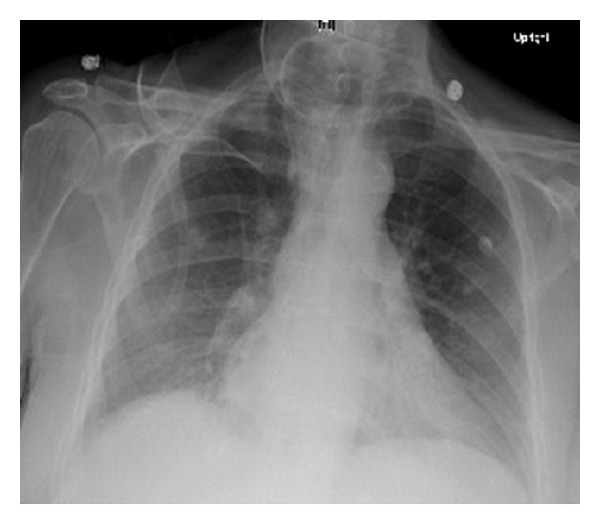
Chest X ray with no abnormal findings.

Laboratory results revealed platelets of 30,000/*μ*L, hemoglobin of 13.9 gm/dL, and white cell count of 8000/*μ*L. Metabolic profile revealed the following: sodium: 141 meq/L, potassium: 2.9 mEq/L, bicarbonate: 31 mmol/L, glucose: 161 mg/dL, AST: 56 IU/L, ALT: 81 IU/L, ALP 187 IU/L, total bilirubin 1.9 mg/dL, and albumin 3.7 g/dL. Serum phosphate, calcium, and magnesium were normal. Serum studies for viral hepatitis, HIV, protein electrophoresis, LDH, haptoglobin, Coombs test, flow cytometry, coagulation profile, B12, and folate levels were normal. Peripheral smear revealed decreased platelets. There was a mild left shift with rare nucleated red blood cells suggestive of some degree of leukoerythroblastosis. Initial chest X-ray was normal (Figure 1). Radiograph of the spine did not reveal any metastatic disease. A CT scan of the abdomen revealed cirrhosis of the liver.

**Figure 2:**
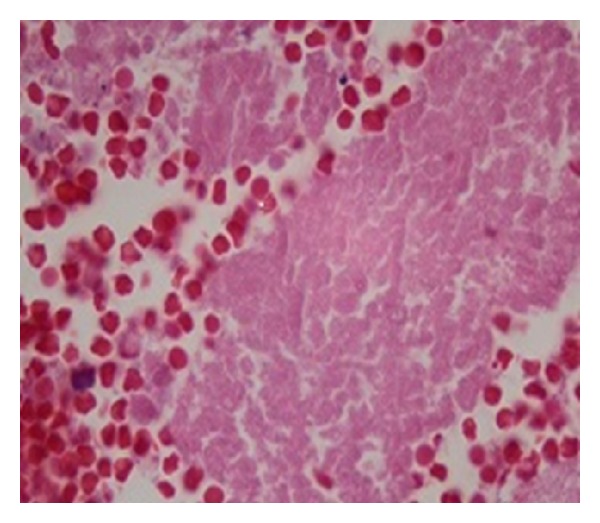
Bone marrow biopsy showing bone marrow necrosis which may be from tumor cells itself or the effect of tumor cells on marrow cells.

**Figure 3:**
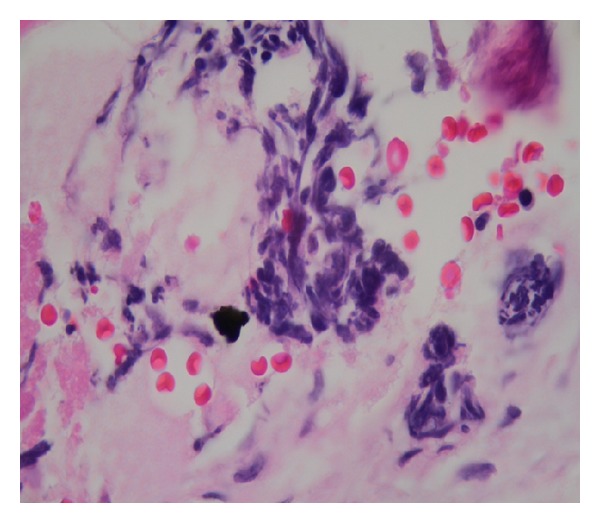
Bone marrow smear showing atypical small cells with sparse acidophil cytoplasm, lobulated and rounded nuclei, and compact chromatin.

**Figure 4:**
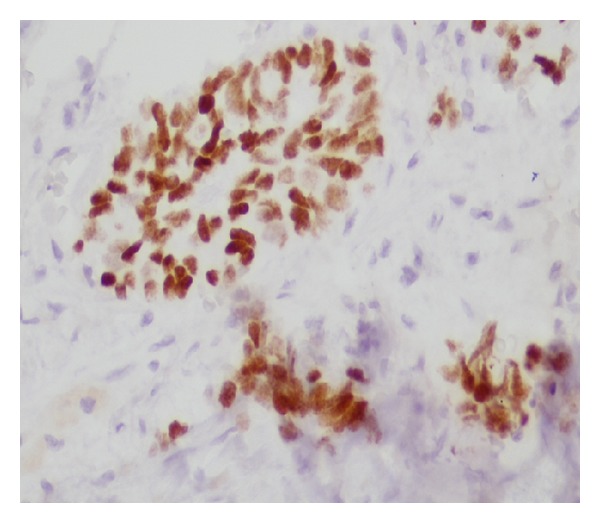
TTF 1 positive staining of the neoplastic cells confirming the cells are of pulmonary origin.

**Figure 5:**
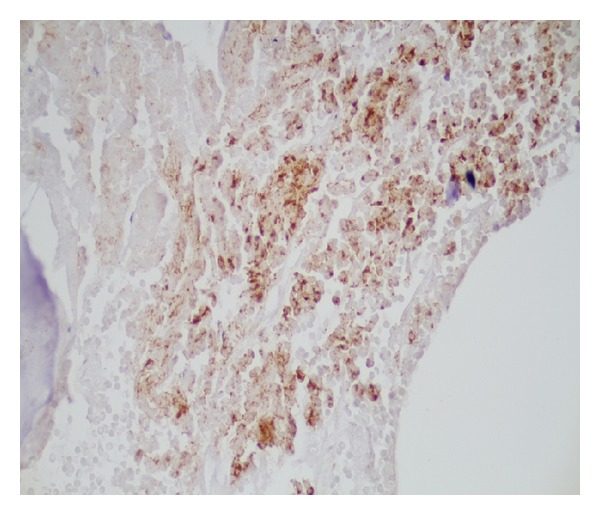
Chromogranin positive neoplastic cells in the bone marrow suggestive of small cell lung cancer.

Initially her thrombocytopenia was attributed to cirrhosis. During the hospital stay the platelet count was rapidly decreasing to reach a nadir of 9000/*μ*L. The patient received multiple platelet transfusions. Meanwhile her hypokalemia was refractory to continuous oral and intravenous potassium supplements (Table 1). A spot urine potassium was high (50 mEq/L) suggesting renal loss. At this point of time her thrombocytopenia was a serious concern. A bone marrow biopsy was performed for evaluation of severe persistent thrombocytopenia. Examination of the bone marrow showed diffuse marrow necrosis with rare focus of viable marrow which was partially replaced by metastatic small cell carcinoma. The necrotic areas of marrow showed nonspecific immunohistochemical staining for cytokeratin and chromogranin with high background which precluded definitive differentiation of necrosis limited to tumor versus total marrow necrosis (Figures 2, 3, 4, and 5). The patient did have a history of smoking.

**Figure 6:**
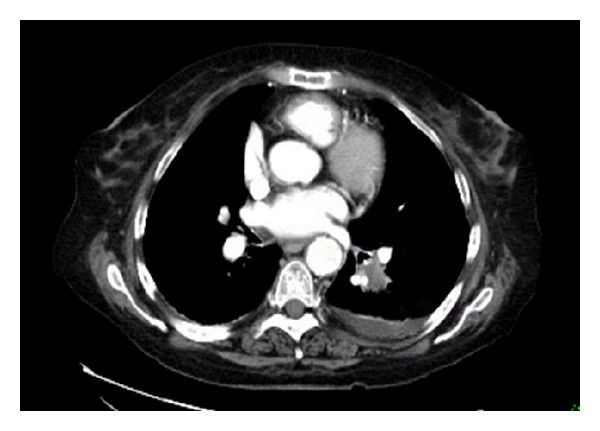
CT scan of the chest showing a spiculated mass in left lower lobe of the lung.

Subsequent contrast enhanced CT scan of the lung showed mediastinal adenopathy with a spiculated left hilar mass (Figure 6). Serum ACTH level was elevated to 106 pg/mL (normal value is less than 47 pg/mL) as was the 24-hour urine free cortisol level (1000 *μ*g, normal value < 100 *μ*g), with a normal renin and aldosterone level. The metabolic findings, high ACTH level with radiologic and histological evidence, made ectopic ACTH syndrome from small cell lung cancer the most likely diagnosis. Interestingly refractory hypokalemia was the only feature in ectopic ACTH syndrome making the diagnosis very challenging. The patient was started on ketoconazole for the treatment of ectopic ACTH syndrome. There was no evidence of tumor lysis syndrome. Eventually the patient's hemoglobin and WBC counts also dropped but to a very less extent with nadir being only 10 gm/dL and 4000/*μ*L, respectively.

The patient was not able to receive standard chemotherapy with carboplatin and etoposide for small cell lung cancer due to severe thrombocytopenia and cirrhosis which are the relative contraindications of the above drugs, respectively. The patient died within several days due to extensive metastatic spread with total hospital course of only 1 week.

## 3. Discussion

Small cell lung cancer is a neuroendocrine carcinoma that exhibits aggressive growth, rapid spread, exquisite sensitivity to chemotherapy, and radiation [4]. Some paraneoplastic syndromes have been associated with small cell lung cancer like SIADH (15%), ectopic ACTH (2–5%), and Eaton Lambert syndrome (3%) [5–7]. Recently paraneoplastic thrombocytopenias have also been described in small cell lung cancer including ITP, TTP, and amegakaryocytic thrombocytopenia [2]. Paraneoplastic syndromes can be recognized without much difficulty in established cases of small cell lung cancer. Very rarely, they can be the manifesting picture of cancer, which poses extreme diagnostic and therapeutic dilemmas. The patient in our case presented with two rare initial manifestations of small cell lung cancer: refractory hypokalemia and isolated thrombocytopenia.

Hypokalemia refractory to large potassium supplements can be one of the features of ectopic ACTH syndrome. Very few cases have been reported describing hypokalemia as an initial presentation of small cell lung cancer. Typical presentation of ectopic ACTH syndrome includes hypertension, hyperglycemia, hypokalemia, and metabolic alkalosis with cushingoid features [1, 6]. All the features may not present simultaneously which can make the diagnosis difficult. Ectopic ACTH from malignant neoplasm has rapid and more aggressive metabolic effects as described in previous studies compared to Cushing's syndrome [8]. Hypokalemia is present due to mineralocorticoid effects of hypercortisolism from ectopic ACTH secretion. The local cortisol conversion to cortisone by the action of 11 beta-hydroxysteroid dehydrogenase is the rate-limiting step for the mineralocorticoid effect of cortisol under normal conditions. When plasma concentrations of cortisol are very high, the action of this enzyme is insufficient (the mineralocorticoid escape phenomenon) and mineralocorticoid effects appear [1]. In this case, the rapid clinical course did not permit pituitary magnetic resonance imaging or a test like inferior petrosal venous sampling (considered as gold standard) for ectopic ACTH-dependent Cushing's syndrome. Ketoconazole has been used to treat Cushing's syndrome by inhibiting adrenal glucocorticoid synthesis. It has been shown to resolve hypokalemia by preventing mineralocorticoid production. Treatment with ketoconazole promotes a palliative hormonal response in more than 50% of patients [9]. However due to rapid clinical deterioration with the condition being diagnosed at the end, ketoconazole was not very effective in our patient.

Thrombocytopenia can be caused by multiple conditions. Less recognized causes include solid malignancies due to either bone marrow metastases or a paraneoplastic phenomenon [2]. The various paraneoplastic conditions include ITP, TTP, myelodysplastic syndrome, and amegakaryocytic thrombocytopenia [2]. Bone marrow biopsy is the key diagnostic step for evaluating thrombocytopenia in cases of unclear etiology. Our patient had severe bone marrow necrosis on marrow biopsy. Bone marrow necrosis is a rare clinicopathologic entity and is relatively unknown and unrecognized. Bone marrow necrosis is defined as, “necrosis of the myeloid issue and the medullary stroma in large areas of the haematopoietic bone marrow” [10]. It was first described by Wade and Stevenson in 1941 [11], in a patient of sickle disease, who had died of cerebral infarction; however it is still an under recognized entity. The incidence of BMN is reported to be in range of 0.37% to 6.5% [12]. It is caused by hypoxemia due to failure of microcirculation. In a recent retrospective study by Janssens et al., in patients with bone marrow necrosis, 90% of them had underlying malignancy [10]. Hence it is important to look for malignancy or marrow metastases in cases of marrow necrosis and probably also stain the marrow with special stains for tumor to detect malignancy. Marrow necrosis was likely from metastatic cells invading the marrow cells in our patient. One of the most common clinical presentations is bone pain. Interestingly severe thrombocytopenia was the predominating feature of bone marrow necrosis in our case compared to anemia or neutropenia.

The association of ectopic ACTH production by SCLC and bone marrow involvement is probably not just a chance association. In the study by Shepherd more than half of their patients with ectopic ACTH also had evidence of bone marrow involvement [6].

## 4. Conclusions

(1) Refractory hypokalemia can be a part of ectopic ACTH syndrome and a high clinical suspicion is necessary to search for an occult malignancy. Failure to recognize ectopic ACTH syndrome can lead to additional morbidity and mortality. (2) Isolated thrombocytopenia can be an initial presentation of solid tumors due to marrow metastases; thus occult malignancy should always be kept in consideration in patients with thrombocytopenia of unclear etiology. (3) Given the high rate of malignancy as an underlying disease association, an extensive search for neoplastic disease is justified whenever bone marrow necrosis is diagnosed.

## Figures and Tables

**Table 1:** Serum potassium values in response to potassium supplements.

	Day 1	Day 2	Day 3	Day 4	Day 5
Potassium level in mEq/L	2.9	3.2	2.8	2.3	2.7
Potassium supplement in mEq	80	80	160	180	160
